# Assessment of Microstructural, Mechanical and Electrochemical Properties of Ti–42Nb Alloy Manufactured by Electron Beam Melting

**DOI:** 10.3390/ma16134821

**Published:** 2023-07-04

**Authors:** Maria Kozadaeva, Maria Surmeneva, Dmitriy Khrapov, Vladimir Rybakov, Roman Surmenev, Andrey Koptyug, Alina Vladescu (Dragomir), Cosmin Mihai Cotrut, Alexander Tyurin, Irina Grubova

**Affiliations:** 1 Physical Materials Science and Composite Materials Centre, Research School of Chemistry & Applied Biomedical Sciences, National Research Tomsk Polytechnic University, 30 Lenina Avenue, 634050 Tomsk, Russia; 2 International Research and Development Center “Piezo- and Magnetoelectric Materials”, Research School of Chemistry and Applied Biomedical Sciences, National Research Tomsk Polytechnic University, 30 Lenina Avenue, 634050 Tomsk, Russia; 3 Sports Tech Research Centre, Mid Sweden University, Akademigatan 1, SE 83125 Östersund, Sweden; 4 National Institute of Research and Development for Optoelectronics INOE 2000, 409 Atomistilor St., 77125 Magurele, Romania; 5 Faculty of Materials and Science Engineering, University Politehnica of Bucharest, 313, Spl. Independentei, 060042 Bucharest, Romania; 6 Institute “Nanotechnology and Nanomaterials”, G.R. Derzhavin Tambov State University, 33 Internationalnaya St., 392000 Tambov, Russia

**Keywords:** implant material, Ti–2Nb alloy, additive manufacturing, mechanical properties, corrosion resistance, β type Ni-free Ti alloy

## Abstract

The β-type Ti–42Nb alloy has been successfully manufactured from pre-alloyed powder using the E-PBF method for the first time. This study presents thorough microstructural investigations employing diverse methodologies such as EDS, XRD, TEM, and EBSD, while mechanical properties are assessed using UPT, nanoindentation, and compression tests. Microstructural analysis reveals that Ti–42Nb alloy primarily consisted of the β phase with the presence of a small amount of nano-sized α″-martensite formed upon fast cooling. The bimodal-grained microstructure of Ti–42Nb alloy comprising epitaxially grown fine equiaxed and elongated equiaxed β-grains with an average grain size of 40 ± 28 µm exhibited a weak texture. The study shows that the obtained microstructure leads to improved mechanical properties. Young’s modulus of 78.69 GPa is significantly lower than that of cp-Ti and Ti–6Al–4V alloys. The yield strength (379 MPa) and hardness (3.2 ± 0.5 GPa) also meet the criteria and closely approximate the values typical of cortical bone. UPT offers a reliable opportunity to study the nature of the ductility of the Ti–42Nb alloy by calculating its elastic constants. XPS surface analysis and electrochemical experiments demonstrate that the better corrosion resistance of the alloy in SBF is maintained by the dominant presence of TiO_2_ and Nb_2_O_5_. The results provide valuable insights into the development of novel low-modulus Ti–Nb alloys, which are interesting materials for additive-manufactured implants with the desired properties required for their biomedical applications.

## 1. Introduction

Implant materials replacing hard tissues must have outstanding mechanical properties and be biocompatible to prevent cytotoxicity and provide long service time. These requirements arise from the need to withstand repetitive loads, resist fatigue, and prevent so-called stress shielding [1]. When a surgical implant replaces a part of the skeleton, it takes on a significant portion of the mechanical load, safeguarding the adjacent bone.

At present Ti-based alloys are the most common materials used in dental and orthopedic implants [2,3]. Unfortunately, most commonly used cp-Ti and Ti–6Al–4V do not adequately match the mechanical properties of the bones being in a way much stronger than natural bones. Ti alloys with the elements like Ta, Mo, Nb, and Zr stabilizing bcc β-phase having mechanical properties much closer to that of the bone have a potential for a good solution. β-Ti alloys have lower elastic modulus compared to conventional α or α–β Ti alloys (<80 vs. 110~120 GPa) [4,5,6,7]. Corresponding field of research for new biocompatible β-Ti alloys is extremely wide with uncountable options even among the binary and ternary compositions forcing researchers to concentrate at certain most promising directions. Significant attention is recently attracted to the binary β-Ti alloys with Nb and Zr. For example, the Ti–Nb system provides a good combination of biocompatibility and low Young’s modulus, leading to the development of many new β-type Ti-based alloy compositions.

The field of metal additive manufacturing (AM) of implants is constantly gaining popularity first of all due to the unprecedented shape freedom it provides and the ability of combining solid and porous sections of implant within the same process [8,9]. Powder bed fusion (PBF) at the moment is the most commonly used AM family, including in particular selective laser melting (L-PBF) and electron beam melting (E-PBF). However, E-PBF has certain advantages over L-PBF because it preheats the powder bed during manufacturing resulting in reduced residual stress in manufactured components [9,10,11]. The development of AM processes for Ti alloys faces multiple challenges, one of them being an anisotropy in the material microstructure, in particular due to the growth of columnar β-grains along the temperature gradients. If not treated properly it leads to significant spatial anisotropy of material properties in the manufactured components such for example the yield and tensile strength. Thus, the development of AM processes for new Ti alloys often targets achieving material microstructures with small-sized equiaxed grains [8,12]. As is common in many AM modalities, this is achieved through alloy composition selection, targeted optimization of process parameters, and post-processing. Modern advances in AM technology, in particular its beam-based representatives such as L- and E-PBF methods also allow for the microstructure tailoring in 3D through manipulating beam scan and energy parameters [13]. The application of such a strategy for controlling microstructure and mechanical properties in β-type Ti alloys is creating a good potential for manufacturing patient-specific implants with optimal properties for in vivo conditions [14]. 

Several efforts have been made in the literature to produce Ti–Nb alloys with optimal mechanical properties using AM [5,15,16,17,18]. Weinmann et al. [15] successfully manufactured Ti–42 wt.% Nb specimens via L-PBF. They reported the production of these alloys with high fidelity and observed mechanical properties. The obtained compressive modulus *K* was determined to be 55–64 GPa. In another investigation by Schulze et al. [5], L-PBF manufactured Ti–42 wt.% Nb alloy has values of Young’s modulus and ultimate yield strength of 60.5 GPa and 683 MPa, respectively. Fischer et al. [18] conducted an in-depth investigation on a metastable beta Ti–26 at.% Nb alloy, which was fabricated in situ using pure titanium and pure niobium powders through L-PBF. The authors revealed that a homogeneous alloy could be achieved with minimal un-melted Nb particles, porosity below 3%, and Young’s modulus of approximately 77 GPa. 

The influence of the spontaneously formed native passive oxide film on titanium (Ti) and its alloys extends beyond determining corrosion resistance and the release of metallic ions; it also affects the biocompatibility of implants [19]. In the case of β-type Ti–xNb alloys, the corrosion behavior and biocompatibility are influenced by the presence of the stabilizing element Nb in the passive film, which consists of a mixture of protective oxides such as TiO_2_ and Nb_2_O_5_ [20,21,22]. In a recent study [23], the corrosion behavior of Ti–Nb alloys containing 5–20 wt.% Nb was investigated in a NaCl solution. The results indicated that the Ti–xNb alloys displayed superior corrosion resistance compared to cp-Ti. Another study [20] revealed the presence of Nb_2_O_5_ on the surface of a Ti–45 wt.% Nb alloy played a role in preventing attack by fluoride ions. Furthermore, this study compared the corrosion behavior of the Ti–Nb alloy with that of a titanium-molybdenum alloy (TMA) and found that the Ti–Nb alloy exhibited better corrosion resistance in fluoride-containing artificial saliva than the TMA samples. Choe et al. [21], observed that the pitting corrosion resistance improved as the niobium content increased from 10 to 40 wt.% in a 0.9% NaCl solution. However, to the best of our knowledge, there have been no studies examining the corrosion properties of additively manufactured Ti alloys with a relatively high niobium content.

In our previous study, the low-modulus metastable β Ti–42Nb alloy was for the first time fabricated using E-PBF. Corresponding process parameters were carefully selected in order to achieve the optimum microstructure and mechanical properties [24]. A processing regime with a beam current of 4 mA, line offset of 100 µm, and scanning speed of 800 mm/s was considered appropriate. Despite of defining the E-PBF processing window for β Ti–42Nb, there is a lack of comprehensive data regarding the functional properties of the alloy preventing further process optimization. Hence, the current work is the second part of an ongoing study estimating the correlation between the microstructure, texture, mechanical properties, and corrosion characteristics of the Ti–42Nb manufactured under this specific processing. The complex approach was applied to study the mechanical properties using three available methods: nanoindentation, compression tests and the ultrasonic pulse-echo technique (UPT). In addition, through determining longitudinal and shear sound velocities we evaluated the elastic constants and moduli as well as the Debye temperature and minimum thermal conductivity, which were not known before. Therefore, the findings may serve as a reference for future process optimization, targeted texture design and manufacturing of isotropic low modulus Ti–42Nb alloy via E-PBF technique. Additional corrosion studies of additively manufactured Ti–42Nb were performed and indicate that this alloy offers excellent corrosion resistance.

## 2. Materials and Methods

### 2.1. Fabrication

The current studies were performed using 10 × 10 × 10 mm samples manufactured from a pre-alloyed powder (AMtrinsic^®^, Taniobis GmbH, Goslar, Germany) containing Ti alloy with 42 wt.% of niobium (Nb). The specimens were manufactured in ARCAM A2 E-PBF machine (ARCAM EBM, GE Additive Company, Mölnycke, Sweden) in a vacuum environment. The powder layer was pre-sintered during the pre-heating stage, with the process temperature initially set between 700 °C and 750 °C. However, due to inadequate process stability and inadequate pre-sintering, preheating had to be increased. Although there is no possibility with existing machine to control powder surface temperature, actual temperature of the powder upper layer was estimated to reach 750–800 °C. A standard raster-type scanning pattern with trace direction rotating 90 degrees for each consecutive layer without additional contour melting was selected. Process regime was chosen basing on the results of our previous work [24]. The chosen layer thickness (*d*), beam current (*I*), beam speed (*v*), and line offset (*h*) as well as calculated Line Energy and Area Energy are presented in Table 1. 

Resulting material porosity and density for the tested specimens were calculated earlier in [24] and are equal to 5.6% and 5.31 g/cm^3^, respectively.

### 2.2. Material Analysis

**Figure 1:**
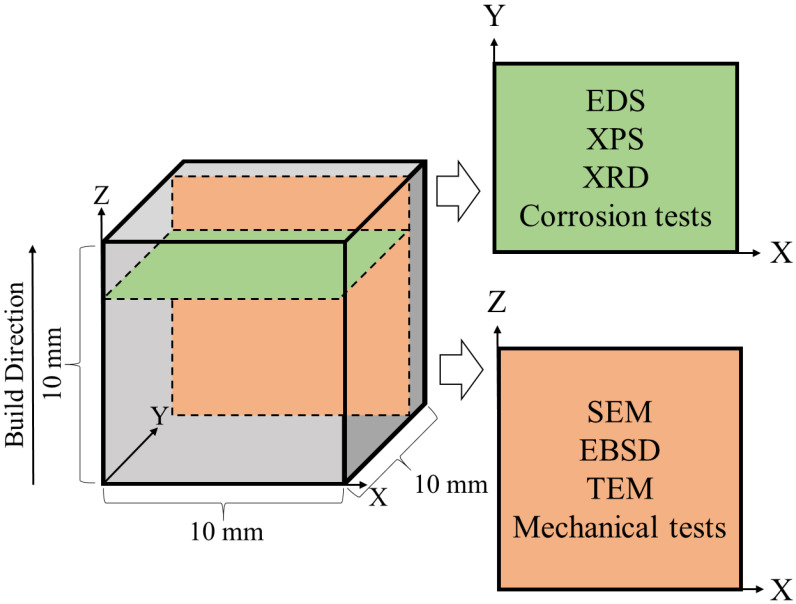
The scheme of the investigation planes.

The mechanical and electrochemical properties of the AM Ti–42Nb alloy were evaluated in the building direction (BD, Z) and in layer plane (X, Y). To accomplish this, the microstructure of the alloy was analyzed using the cuts in two planes as illustrated in Figure 1. Cut planes were offset few mm from the sample surfaces to assure that we study the bulk material properties avoiding the influence of surface effects. 

X-ray photoelectron spectroscopy (XPS) was employed to analyze composition of the alloy in the XY plane. A Thermo Fisher Scientific XPS NEXSA spectrometer (Thermo Fisher Scientific, Waltham, MA, USA) equipped with a monochromated Al Kα Alpha X-ray source operating at 1486.6 eV was used for this purpose. The XPS survey spectra were obtained from a surface area of 400 µm^2^ with a pass energy of 200 eV and an energy resolution of 1 eV. High-resolution spectra were also collected with a pass energy of 50 eV and an energy resolution of 0.1 eV. The flood gun was utilized to compensate for charging. For XPS tests approximately 3 mm of material was polished off the specimen using silicon carbide discs of 230, 400, 800, 1200, 1500, and 2000 grit.

The specimens phase composition was also determined in XY plane by XRD–6000 diffractometer (Shimadzu Corporation, Japan) with CuKα radiation (λ = 0.154 nm) in the 2θ range from 30° to 145° with a step size of 0.02°/2θ at 40 kV and 30 mA. Microstructural properties, such as crystal structure parameter (*a*), dislocation density (δ) and microstress (ε) were calculated basing on X-ray line profile analysis using the Stokes-Wilson equation [25,26]. The Debye–Scherrer formula was used for crystallite size (*D*) estimations [27,28]. The texture coefficients (*TC_hkl_*) were determined with the formula described in [29,30], which uses the relative intensity taken from XRD analysis and the JCPDS database intensities of the diffracted from (*hkl*) planes for the bcc β phase of Ti.

To characterize the microstructure of the specimen in XZ plane scanning electron microscopy (SEM) and electron backscatter diffraction (EBSD) measurements were performed using a field emission scanning electron microscope Tescan MIRA 3 LMU (TESCAN ORSAY HOLD-ING, Brno, Czech Republic), equipped with Oxford Instruments Nordlys EBSD detector and Oxford Instruments Ultim Max 40 EDX detector (Oxford Instruments, High Wycombe, UK). The EBSD mapping was conducted with a scanning step size of 0.8 μm. The specimen for EBSD was polished with silicon carbide discs of 230, 400, 800, 1200, 1500, and 2000 grit for 2–5 min. Then the specimens were finally polished with water based, polycrystalline diamond suspension (1 μm). The grain size distribution and aspect ratio were obtained by Equivalent Circle Diameter method using Oxford Instruments Aztec software (version 3.1, Oxford Instruments, High Wycombe, UK), enabling automatically recognizing grain boundaries and calculating grain parameters.

Thin foils for transmission electron microscopy (TEM, JEOL JEM-2100F, Tokyo, Japan) studies were prepared by ion milling using the Ion Slicer EM-09100IS (JEOL Ltd., Tokyo, Japan).

### 2.3. Nanoindentation, Compression and Ultrasonic Tests

**Figure 2:**
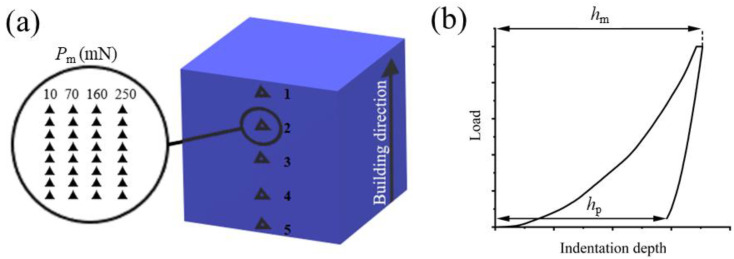
Schematic nanoindentation pattern on the specimen surface (**a**) and schematic loading-unloading curve showing maximum indentation depth and residual deformation (**b**).

In our recently published research, we have reported results from indentation tests for the specimens of Ti–42Nb alloy produced by the E-PBF method [24]. A well distinguished recurrent near-periodic pattern in nanohardness (*H*) and Young’s modulus (*E*) values with the period of about 50 µm have been identified along the BD. In present research further study of hardness and elastic modulus at different penetration depths was carried out to provide a more comprehensive understanding of bulk mechanical properties and behavior of studied alloy. Hysitron Nanotriboindenter TI-950 with much finer spatial resolution was used as an indentation tool. Series of indents were made in the areas 1–5, with a distance of 500 µm between the clusters (Figure 2a). Each nanoindentation cluster was centered at the middle of corresponding solidified layer. Different peak loads *P*_max_ from 10 mN to 250 mN was used to assess the behavior of the alloy during both loading and unloading at different depths (Figure 2b). The peak force was held for 10 s before unloading to check the creep behavior.

Superelastic depth recovery was calculated from the indentation curves (Figure 2b) using following Equation (1):

(1) $$\eta_{h}=\frac{h_{m}{–h}_{p}}{h_{m}}$$

where *h_m_* is the maximum indentation depth and *h_p_* is permanent deformation depth. 

Compression tests were performed using a universal testing machine INSTRON 3369 (Instron Deutschland GmbH, Darmstadt, Germany). Tests were conducted using a displacement rate of 0.5 mm/min. The failure strain was set at 30% of the specimen height. For these tests three samples with dimensions 2.37 × 2.25 × (2.24 ± 0.01) mm were prepared by electrical discharge machine (EDM).

The standard UPT method was used to determine the velocities of longitudinal and shear waves in the material. Measurements were performed using ultrasonic thickness gauge Olympus 38 DL PLUS (USA) with longitudinal wave and shear wave probes, using ultrasonic coupling gel. Acquired values of sound velocities were used to calculate elastic constants, Poisson’s ratio (ν), hardness (*H_u_*), elastic (*E_u_*), shear (*G*), and bulk (*B*) moduli of the alloy, using formulas outlined in [31,32,33]. The Anderson formula was utilized to determine the Debye temperature (θ*_D_*) [34]. The Liu relation was employed to calculate minimal conductivity [35].

### 2.4. Electrochemical Behavior Studies

To evaluate the corrosion resistance of the new alloy, the polarization resistance technique was employed. Corresponding potential difference was in the range of ±200 mV vs. OCP (open circuit potential), and Tafel plots with a scan rate of 0.167 mV/s were recorded. The first step in the measurement protocol is achieving the open circuit potential stabilization (in our case—6 h of exposure). Corrosion tests were performed using a Potentiostat/Galvanostat (PARSTAT 4000, Princeton Applied Research, Oak Ridge, TN, USA) coupled with a Low Current Interface (LCI, Princeton Applied Research, Oak Ridge, TN, USA). The potentiodynamic polarization curves were acquired using VersaStudio software. Electrochemical cell with double glass wall (temperature stabilization jacket), a saturated calomel reference electrode (SCE), a platinum recording electrode (CE) and the working electrode (WE) was employed. New Ti–42Nb alloy was used as working electrode. Tests were carried out in simulated body fluid (SBF) at the human body temperature (37 ± 0.5 °C), using external thermostat stabilizing the inner temperature by circulating external liquid between the double walls of the cell. The experiments were performed in triplicate. The chemical composition of employed SBF is given in the Table 2.

## 3. Results and Discussion

### 3.1. Material Microstructure

**Figure 3:**
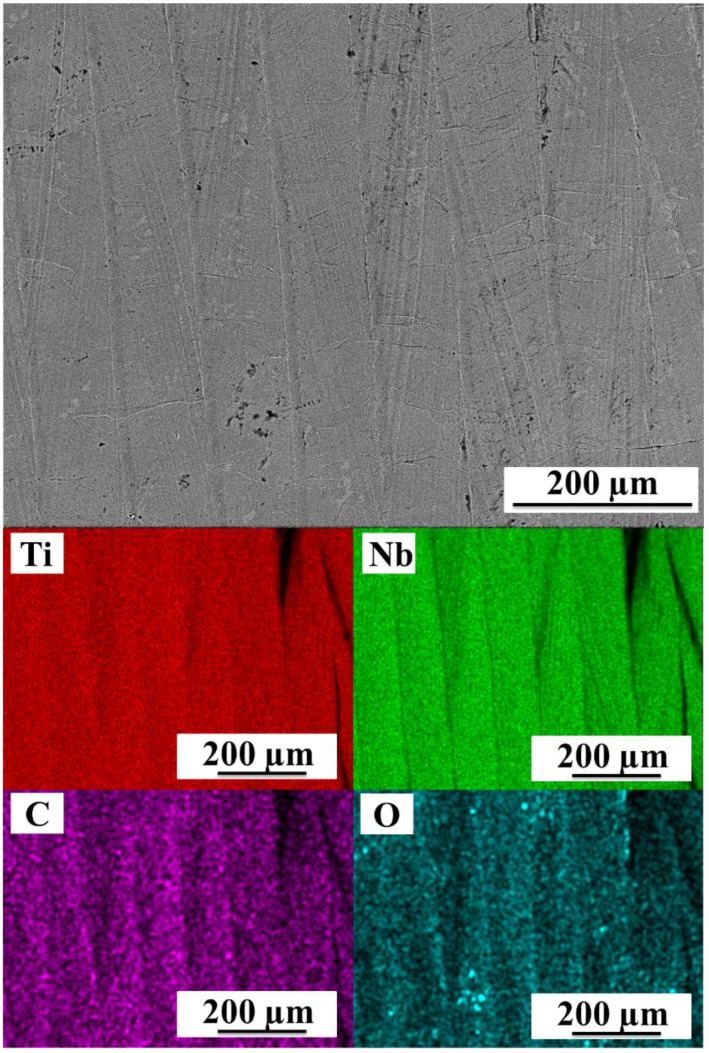
BSE-SEM image of the specimen (the contrast is intensified) and EDS composition mapping distribution in the layer (XY) plane after polishing. Ti, Nb, C and O density maps are in red, green, violet, and blue color, respectively.

The SEM morphology and EDS elemental mapping images of the same region of the Ti–42Nb alloy in the layer (XY) plane after polishing are shown in Figure 3. There are a small number of pores without obvious non-melted areas, confirming that processing parameters allowed for the fabrication of fully dense specimens. The high energy density of the beam used during processing caused an extension of the melt pool cross-section normal to scan line, particularly pronounced at the beam turning points. And although ARCAM firmware automatically increases the beam speed near the turning points [36] there may be a need to further optimize the scanning strategy. Based on the EDS composition mapping of Ti, Nb, C, and O, it can be concluded that these elements are uniformly distributed throughout the material. 

**Figure 4:**
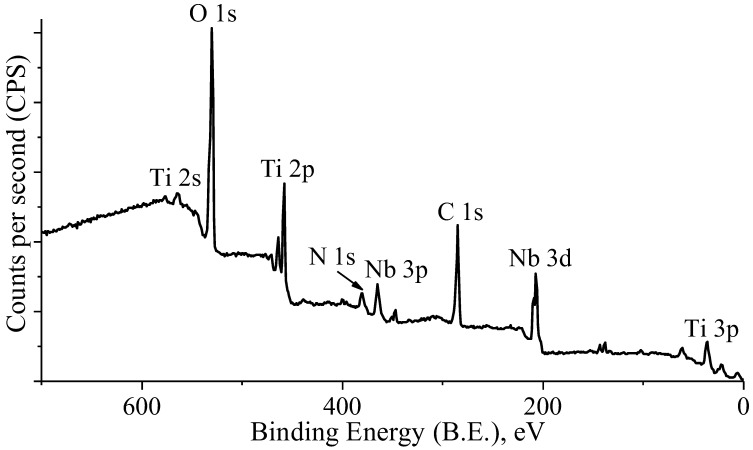
XPS survey spectra of the Ti–42Nb alloy surface in the layer (XY) plane.

The properties of Ti surfaces are significantly dependent on the Ti oxide film that forms naturally upon exposure to air. Remarkable chemical inertness, corrosion resistance, and repassivation ability of Ti and its alloys can be attributed to the chemical stability and structural integrity of this oxide film [37]. XPS analysis was performed to obtain a more precise quantitative evaluation of the elemental composition of the specimen’s surface. The XPS survey spectra of the Ti–42Nb specimen surface are presented in Figure 4. The presence of oxygen and nitrogen was expected since these elements are commonly adsorbed on Ti surfaces. Presence of carbon most probably is due to the contamination by some organic compounds. A dominant doublet peak at ≈458 eV and ≈464 eV on the survey scan can be attributed to Ti oxide [38,39].

The composition of the alloy surface, as determined by quantitative XPS analysis, presented in Table 3, reveals the measured binding energies of Ti, Nb, and O. It should be noted that the lower levels of Ti and Nb compared to the literature values suggest a thicker oxide film [40,41]. Peng et al. [41] found that increasing the oxide film thickness on the surface of Ti–Nb alloy modified by electrochemical treatment from 70 to 250 nm results in a decrease in measured Ti content from 20 at.% to 13 at.% and Nb content from 9 at.% to 5 at.%. The ratio [Ti]/([Ti] + [Nb]) in the passive film is lower than that in the initial powder (0.61 vs. 0.74), while the [Nb]/([Ti] + [Nb]) ratio is higher (0.39 vs. 0.27). An increase in Nb and Nb_2_O_5_ content on the surface should lead to improved biomaterial performance of the Ti–42Nb alloy [40,41].

**Figure 5:**
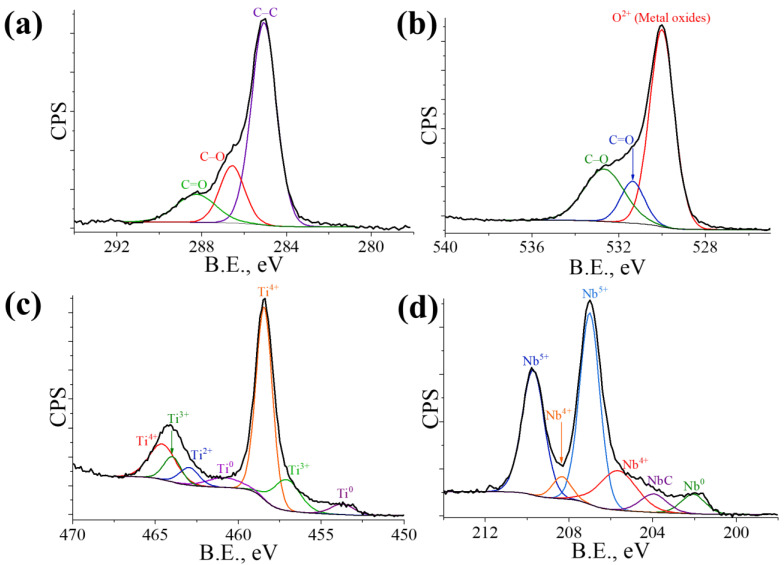
Automatically deconvoluted XPS spectra of the Ti–42Nb alloy surface in the layer (XY) plane: C 1s (**a**), O 1s (**b**), Ti 2p (**c**), and Nb 3d (**d**).

Figure 5 presents high-resolution spectra of the main elements. The C 1s photoelectron spectrum (Figure 5a) shows distinct ‘shoulders’ corresponding to the presence of aliphatic hydrocarbon contamination (≈285 eV) and functional groups of C–O and C=O at ≈286.5 eV and 288.3 eV, respectively [38,42]. The low energy O 1s line (Figure 5b) indicates the presence of covalently bonded oxygen atoms in the Ti (Ti–Nb) oxide phase, mostly originating from lattice oxygen of Ti (Nb) oxides [42]. The high energy O 1s lines reveal the existence of Ti (Nb)–OH groups and oxidized hydrocarbon contamination on the investigated surfaces. Ti 2p spectrum (Figure 5c) exhibits four doublets, which signify the presence of Ti^0^ (metallic state), Ti^2+^ (TiO), Ti^3+^ (Ti_2_O_3_), and Ti^4+^ (TiO_2_) [38,39,43,44].

The Nb 3d region of the Ti–42Nb alloy spectrum exhibits two distinct peaks at 209.7 eV and 207.0 eV (Figure 5d), which correspond to Nb 3d_3/2_ and Nb 3d_5/2_, respectively. These values are consistent with the standard values for Nb_2_O_5_ [39,45,46]. In addition, the Nb spectrum shows the presence of metallic Nb^0^ (peak at 202.1 eV), a peak at 205.6 eV that may correspond to NbO_2_ or Nb-oxicarbides (NbC_1−x_O_x_), and a peak at 203.9 eV of Nb–C [46,47]. The small amount of NbC is in agreement with the results of our previous study of the virgin Ti–42Nb powder used and E-PBF fabricated alloys produced using various manufacturing parameters [24].

Therefore, it can be concluded that the passive film formed on the Ti–42Nb alloy is mainly composed of TiO_2_ and Nb_2_O_5_, with small amounts of NbC and other Ti (Nb) oxides. The films of such composition are characterized by superior corrosion resistance, when compared to Ti–6Al–4V and Ti–Mo alloys [20,21,22]. In addition, according to Wang et al. [39], such oxide film, consisting mainly of TiO_2_ and Nb_2_O_5_, has an inhibitory barrier effect on the release of internal metal ions and exhibits excellent in vitro biocompatibility.

**Figure 6:**
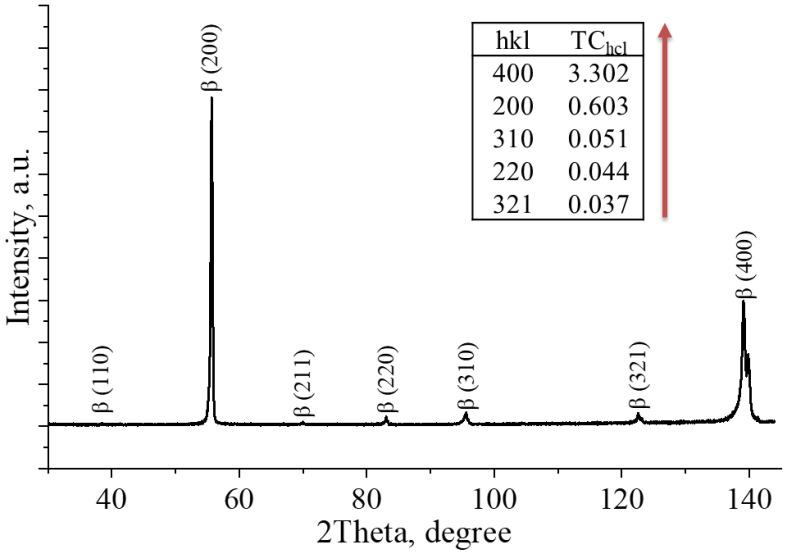
XRD pattern of the as-manufactured by E-PBF Ti–42Nb specimen in layer (XY) plane.

The XRD pattern of the Ti–42Nb specimen, Figure 6, shows that the only present phase is the β-phase, indicating that the melt pool cooling rate corresponding to the selected scan speed of 800 mm/s and chosen beam energy is high enough to maintain a metastable β-phase through the solidification. According to literature [48,49], the β-phase Ti alloys have good biocompatibility and show most appropriate mechanical properties for implant applications. 

Texture coefficients calculated for crystal planes (200), (220), (310), (321), and (400), are shown in the insert in Figure 6. The texture coefficient reflects the texture of a particular diffraction plane, and a deviation from the standard sample values signify a preferred growth pattern. The crystallographic texture for the XY plane cut of the Ti–42Nb specimen indicates that the preferred orientation is the (400) crystal plane, whereas for the XZ plane cut, as reported in [24], the preferred orientation is the (220) plane.

The corresponding calculations of crystal structure parameter, dislocation density, microstresses, and crystallite sizes were performed for further analysis of microstructural properties. The calculated lattice parameters listed in Table 4 are in line with that of β-metastable Ti–Nb alloys measured by other researchers [48,50]. However, using only this information would not be enough to determine the exact phase composition of the specimens since the α″-phase can also be found in Ti–Nb alloys (as it was shown for the alloys with a Nb content of 27 at.%). To authenticate the composition, EBSD and TEM analyses were carried out.

**Figure 7:**
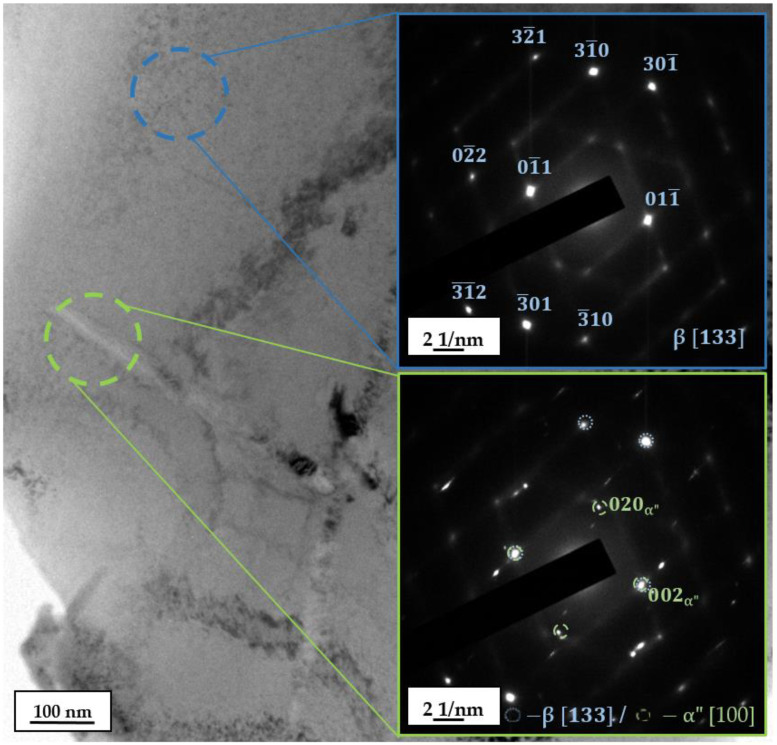
Detailed microstructure of Ti–42Nb specimen with needle-like α″-Ti phase in β-Ti matrix (TEM).

The microstructure of the specimens under study at much smaller dimensional scales was investigated using the TEM method. The light field image of the Ti–42Nb microstructure, along with its corresponding microdiffraction patterns (Figure 7), revealed the presence of needle-like structures (0.3–0.7 μm) of the precipitated α″-Ti phase (orthorhombic crystalline lattice, *Cmcm*), which were distributed throughout the β-grains. These observations support the results from our previous study [24], where different types of martensitic α″-Ti phase were detected.

**Figure 8:**
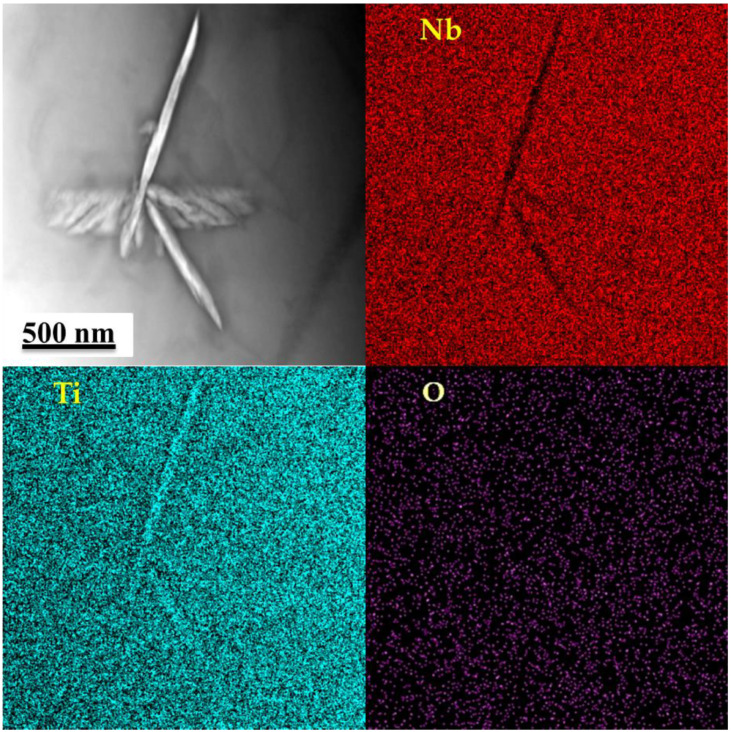
Typical BF TEM image and corresponded mapping of the elemental composition Nb-L, Ti-K and O-K.

A typical HTEM image of the Ti–42Nb specimen microstructure, along with the corresponding EDS mapping of the elemental composition (Figure 8), indicate that the β-Ti phase contains more Nb than the α″-Ti phase. This is attributed to the role of Nb as a β-stabilizer, which leads to greater dissolution in the β-Ti phase compared to the α″-Ti one. In summary, the microstructure of the studied alloy manufactured with chosen process parameters was found to be almost entirely composed of the bcc β-phase, with only a small amount of the martensite α″-phase. The formation of the α″-phase is associated with the high rates of strongly localized heating by the electron beam and subsequent rapid cooling, as described in the literature [51].

**Figure 9:**
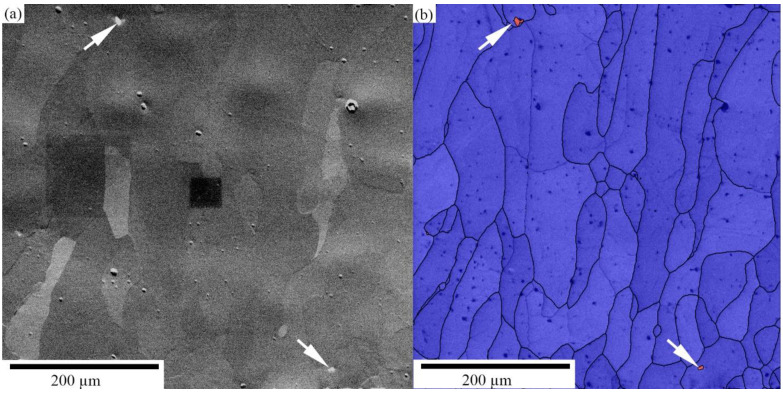
Microstructure of Ti–42Nb: BSE-SEM image (the contrast is intensified) (**a**), band contrast image (**b**).

The microstructure of the E-PBF manufactured Ti–42Nb specimens was also investigated by EBSD to describe the grain size and aspect ratio distribution, texture, and to estimate the impact of microstructural features on the mechanical properties of the alloy. According to BSE-SEM image presented in Figure 9a, no distinct microstructure was observed. Small numbers of spherical pores were detected. The red dots observed on the band contrast image, presented in Figure 9b (indicated by the arrows), corresponding to light-grey dots seen in the SEM image (marked by arrows) are most probably NbC inclusions. We have assessed average concentration of such inclusions to be less than 1%. Most probably they are present in the powder despite of a good quality of pre-alloyed powder.

**Figure 10:**
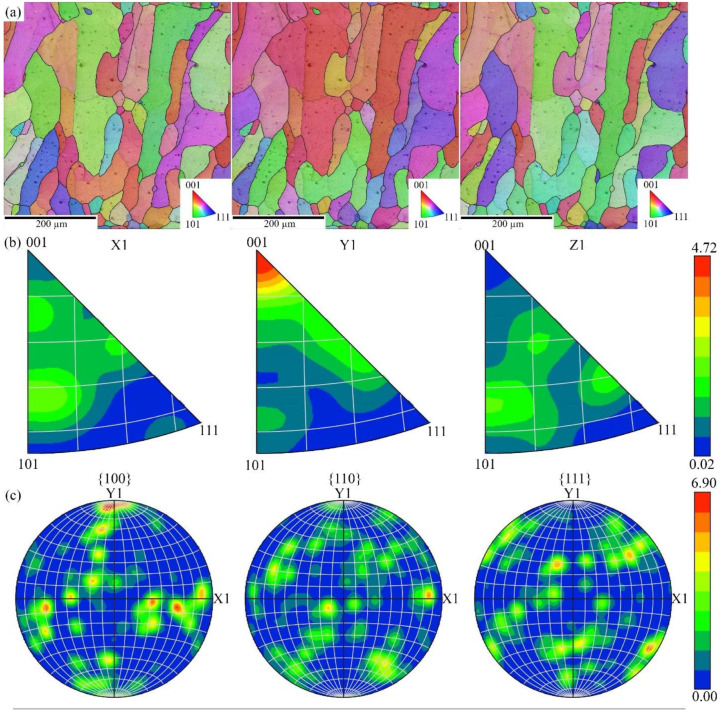
Inverse pole figure maps (**a**), Inversed Pole Figures (**b**), Pole Figures (**c**).

Inversed pole figure (IPF) maps performed for the same area presented in Figure 10a, revealed that the microstructure consists of epitaxial grains, with no preferred crystallographic orientation. The maximum intensity of the pole density of the inversed pole figures, Figure 10b, in the longitudinal cross-section was observed for Y1 plane indicating the presence of (100) texture. Low maximum intensity (4.72) indicates that observed texture is close to isotropic. However, pole figures, Figure 10c, revealed no recognizable pattern of any texture although the presence of strong (100) texture, which is quite typical for AM Ti alloys.

The elastic modulus of Ti alloys may have strong crystallographic orientation dependence, which is also not uncommon for additively manufactured specimens. For example, the elastic modulus of the single crystalline β-Ti alloy Ti–30Nb (at.%) alloy (equivalent to Ti–45Nb wt.%), grown by the floating-zone technique, shows pronounced anisotropy, with the highest value of the elastic modulus about 91 GPa along orientation <111> and the lowest value about 39.5 GPa along orientation <100> [52]. A single crystal of Ti–15Mo–5Zr–3Al (wt.%) grown using an optical floating-zone apparatus with two-phase radiofrequency electromagnetic heating shows pronounced anisotropy and revealed the highest value of elastic modulus about 120 GPa along the <111> orientation and the lowest value about 44.4 GPa, along the <001> orientation, determined from ultrasonic sound velocity measurements of a cubic specimens [53].

Consequently, it is possible to reduce the elastic modulus of β-type Ti alloys by manipulating their crystallographic texture. For example, a Ti–15Mo–5Zr–3Al alloy fabricated through laser powder bed fusion (L-PBF) using a bidirectional (zigzag) scanning approach exhibited distinct crystallographic textures with preferred orientations of <100> and <110> along the BD. As a result, the alloy demonstrated *E_x_* = 68.7 GPa and *E_y_* = 99.6 GPa. [54]. LPBF- manufactured Ti–42Nb (wt.%) specimens demonstrated a microstructure with an intense <100> fiber texture parallel to the BD with *E_x_* = 44 GPa, *UTS*_x_ = 720 MPa and *E_z_* = 51 GPa, *UTS*_z_ = 676 MPa [55]. It has been shown that Ti–42Nb (wt.%) alloy manufactured by laser-directed energy deposition (LDED) process contained large elongated β-columnar grains oriented parallel to the BD (Z-axis) [56]. It was suggested that parent grains exhibiting a favorable crystallographic orientation (<100> direction aligned with the BD) growing faster. Hence, these grains become more prominent and larger, leading to anisotropic mechanical properties due to their intense <100> fiber texture. This difference resulted in the variation of mechanical properties: *E_x_* = 59.4 ± 3.0 GPa and *E_z_* = 47.9 ± 3.9 GPa, σ_0.2x_ = 735 ± 22 MPa and σ_0.2z_ = 715 ± 41 MPa, *UTS_x_* = 771 ± 22 MPa and *UTS_z_* = 718 ± 42 MPa. Thus, it can be concluded that the anisotropic texture with columnar grains must be avoided because it promotes the anisotropy of mechanical properties that may threaten the integrity of the additively manufactured samples with complex shape during life cycle. From this point of view, hardly distinguishable texture observed for alloy in the current research is one of the positive features of the Ti–42Nb alloy. 

**Figure 11:**
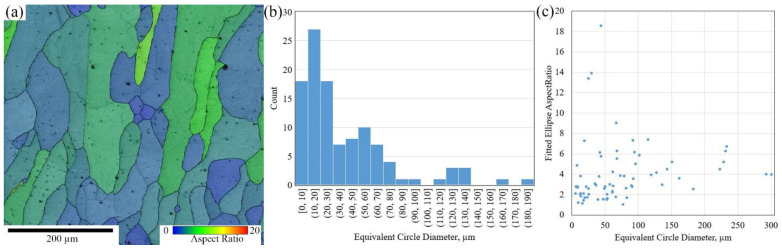
EBSD based grain size analysis: Grain aspect ratio map (**a**), Grain size distribution diagram (**b**), Aspect ratio diagrams (**c**).

In order to more accurately describe the shape and size of the grains, the aspect ratio distribution (relationship between the larger dimension of a grain *R_max_* and the smaller one *R_min_*) and their size distribution were analyzed. Figure 11 presents color mapping of the grain aspect ratio, and grain size and aspect ratio distribution diagrams. The grain size distribution presented in Figure 11b follows a lognormal distribution. Our data show that majority of the grains have equiaxed or distorted- equiaxed shape elongated in the direction of temperature gradient. The majority of the grains have an equiaxed shape, i.e., their aspect ratio is below 3. It is known that a fine equiaxed grain structure better accommodates strain and prevents cracking, and such microstructure is preferable [57]. 

The formation of equiaxed grains as it is commonly supposed is a secondary process that occurs in a supercooled melt pool. The measured average grain size for the chosen area was about 40 ± 28 µm. Some of the columnar grains with an equivalent diameter of about 300 µm were also noted (their aspect ratio was about 4, Figure 11c). In addition, some grains with a shape factor of more than 18 were also observed (their diameter was less than 50 µm). The equiaxed fine grains are associated with the phase transition, which is known as the Columnar to Equiaxed Transition (CET) phenomenon. The grain size of an alloy directly impacts its mechanical properties. The relationship between the average size of crystallites (or grains), the number of dislocations present at grain boundaries, and the yield strength is described by the Hall-Petch dependence. Large grains demonstrate plasticity typically driven by dislocations. Dislocation densities inside grains, except grain boundary dislocations, increase with grain size [58]. The dependency of tensile mechanical properties on the grain size of steel was demonstrated in [59]. When the grain size was increased 10 times, yield and ultimate tensile strengths were decreased from 410.0 MPa and 725.0 MPa to 232.5 MPa and 517.0 MPa, respectively. With the increase of grain size, deformation twins become easy to form. 

As it was demonstrated elsewhere, Nb concentration in Ti–Nb influences the texture of an alloy and its mechanical properties [60]. Analysis of a functionally graded Ti–Nb alloy fabricated by the DED-CLAD^®^ process revealed that Ti–25%Nb alloy consists of large equiaxed grains, while Ti–75%Nb consists of fine equiaxed ones, and Ti–50%Nb contained equiaxed grains with larger size. Tensile tests demonstrated that an increase of Nb content from 5 to 40% resulted in the decrease of the elastic modulus from 99 GPa to 58 GPa, respectively. The further increase of Nb content led to Young’s modulus increasing up to 70 GPa. The alloys with Nb concentration up to 25% demonstrate microhardness 40% greater than that of the alloys with higher Nb content. It is suggested that such growth in the microhardness is due to the presence of a small but increasing fraction of the ω-phase.

The aforementioned presence of α″-phase may influence the mechanical properties of an alloy. The impact of microstructural features on mechanical properties was thoroughly investigated for Ti–15Nb (at.%) alloy containing α″+β phases [61]. During the heat treatment at certain temperature the orthorhombic α″-phase begins to transform to β-phase. During such transformation a growth of β-phase into α″ region happens with the diffusion of Nb atoms away from the α″/β interface and into the growing β phase. The hardness in the zones of α″ to β phase transition (the pre-transformation zone) is commonly about 25% lower as compared to the unaffected area. In the zones, where the transformation is completed, the hardness becomes almost 150% greater that can be explained from the increasing volume fraction α″-martensite that forms from β-phase during cooling. The hardness of the quenched alloy (the post-transformation zone) containing 100% β-phase becomes lower that could be explained by the grain size growth in β-phase. However, the microhardness of the specimen containing α″ + β phases is only slightly lower than microhardness of a specimen containing only β-phase. 

The formation of α″-martensite was noticed for Ti–xNb–4Zr–8Sn alloy when Nb content was less than 24 wt.% [62]. The increase of the Nb content up to 28 wt.% and 36 wt.%, resulted in the formation of an equiaxed grain structure consisting of a single β phase. However, mechanical properties of an alloy with 24 wt.% of Nb demonstrated elastic modulus of only 45 GPa, whereas for the samples with 16 wt.% and 36 wt.% of Nb with different microstructures has higher elastic modulus of about 70 GPa. A cold rolled and solution treated Ti–Nb alloys manufactured with different Nb content revealed different microstructure with different mechanical properties [63]. Ti–30Nb alloys exhibited a single β phase and Young’s moduli in the range of 45–67 GPa, while Ti–20Nb alloys were composed of β and α″ phases and demonstrated elastic modulus of about 49–118 GPa. Thus, the presence of α″-phase may increase Young’s modulus values. The average grain size of β-phase in Ti–20Nb alloys increased significantly with the solution temperature, while Ti–30Nb specimens exhibited a trivial dependence. 

In summary, different factors influence microstructure pattern formation and microstructural features may have a complex effect on mechanical properties. Columnar grains within the (100) direction that are typical for AM alloys and results in anisotropy of mechanical properties were not found in the E-PBF manufactured Ti–42Nb alloy. Instead, epitaxially grown fine equiaxed and elongated equiaxed β-grains with the weak texture were observed. It suggests that the mechanical properties of the material may be isotropic, which was further investigated and is reported in the following section. The average grain size was about 40 ± 28 µm. However, several long (columnar) grains were also detected. Large grains typically corresponded to alloys with low modulus, while fine equiaxed grains are considered as more preferable for good mechanical properties as they accommodate strain and prevent cracking. The presence of some α″-phase was observed, but the impact of this phase on the mechanical properties of β-alloys is quite challenging to predict. Consequently, direct measurements of mechanical properties of the samples were performed.

### 3.2. Mechanical Properties

#### 3.2.1. Nanoindentation Testing

**Figure 12:**
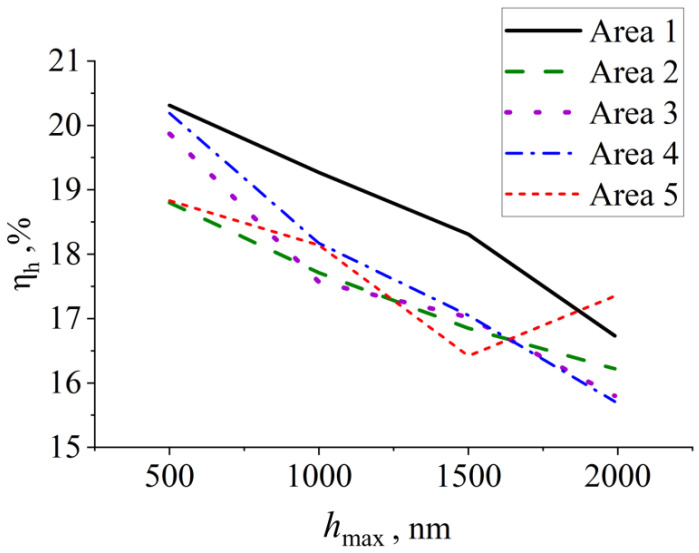
Depth recovery vs. maximum indentation depth along the indentation trajectory.

Nanoindentation tests were carried out to identify the elastic behavior of the alloy at the scales allowing to attribute them to different microstructure patterns. Depth recovery (η_h_) results for all tested areas are presented in Figure 12. The depth recovery values varied from 15.71% to 20.31%. Low η_h_ values indicate non-superelastic behavior of the alloy, while superelastic alloys generally have high ones [64]. For example, Zhou et al. [65] obtained values above 50% for Ti–20Zr–3Mo–3Sn superelastic alloy using a different indenter type probing grain size effect on micromechanical properties.

As it was expected, all curves depth recovery decreases with the depth for all tested areas. This is due to permanent deformation gradually taking over the elastic one. The depth recovery values vary among the indentation Areas not more than 2 units at each depth.

**Figure 13:**
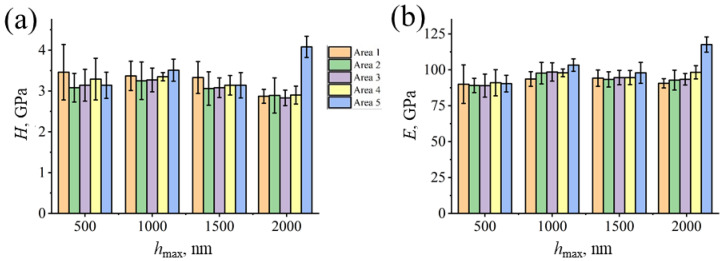
Hardness (**a**) and Young’s modulus (**b**) histograms upon maximum indentation depth in accordance with indentation areas. Corresponding values, presented as clusters of color-coded bars are measured at 500, 1000, 1500 and 200 nm penetration depth.

Investigation of the hardness H and Young’s modulus E with applied force P_max_ = 10 mN (h_max_ = 380 ± 29 nm) resulted in average values of 3.2 ± 0.5 GPa and 89.9 ± 8.3 GPa respectively, whereas the previous research resulted in 3.0 ± 0.8 GPa for H and 68 ± 10.1 GPa for E [24], which as it was mentioned earlier, are affected by both texture and grain size formed during the crystallization of melt pools. Mean values of H and E obtained at different areas with different maximum indentation depths were calculated in the present study and are shown in Figure 13. No significant variations of values were observed along the indentation trajectory at different indentation depths. Except for the values in Area 5, which is located at the bottom of the sample, where the initial layers were melted. Due to the better heat transfer at the bottom layers than at the top layers the melting process may result with different microstructure and micro voids absence.

It is known that average grain size strongly influences the hardness of alloys [66]. The same time indentation measurements data should be treated with care. For example, the grain to the indent size relation is a crucial factor affecting the extracted hardness values, which is known as the indentation size effect. Moreover, indenter type can also play a certain role. For the polycrystalline materials, on the contrary, the hardness typically grows with the indentation depth because of the grain boundary effect [67]. Depending on the indenter and indent size, polycrystalline materials with average grain sizes about 80 µm or larger, the recovered hardness may decrease monotonically with the indentation depth. For smaller grain sizes, the hardness can increase with increasing indentation depth. In general, for small indentation depths, the flow stress is governed by the continued nucleation and activation of new sources, as the grains are considered as single crystals. With well-developed grain structure having relatively large grain and distinct grain boundaries, nanoindentation can produce quite different results depending on particular location of the indent. 

As it was mentioned, no preferred crystallographic orientation in tested Ti–42Nb samples was observed by EBSD and the majority of grains are equiaxed or near-equiaxed. The same time additively manufactured materials can have varying microstructure throughout the sample. It may be due to the temperature gradients in the sample, different cooling rates due to the specifics of beam scanning strategy and heat transfer properties of particular sample. All of these are characteristic to beam-based AM, including E-PBF used in current studies. 

Therefore, it was crucial to assess if there is a non-uniformity of mechanical properties through the volume of a given E-PBF sample. Nanoindentation is one of the methods quite suitable for such analysis. As it was already known that the sample had a recurring pattern of mechanical properties [24], it was possible to design a less dense indentation pattern. And it was confirmed, that mechanical property parameters recovered from nanoindentation experiments at different sample locations are similar, and depth recovery values dispersion does not exceed 2% at all depths, presumably due to similar in size grains being probed.

Previously, it was found by Körner et al. [68] that equiaxed-dominant grain structures are superior to columnar structures in terms of mechanical properties. It was shown, that processing parameters during E-PBF have a strong impact on the resulting microstructure of an alloy. Corresponding data on the microstructure acquired for the Ti–42Nb alloy indicate that it has an equiaxed-dominant grain structure. 

#### 3.2.2. Compression Testing 

Since for load-bearing biomaterials, the preservation of structural integrity under compressive forces is of paramount importance, a series of compression tests was performed to ensure that the alloy is adequate and fit to be used in manufacturing bone implants. These showed a consistent ultimate compressive strength value of 629 ± 29 MPa and at least 26% strain, which prove good overall structural integrity and homogeneity of the alloy. The specimens performed as it should be for a ductile material strong enough to support a human bone, which tops at the ultimate strength of about 221 MPa [69]. Simultaneously with high enough ultimate compressive strength needed materials should show the yield strength close to that of a bone. It is known, that metastable β-Ti alloys commonly have relative low yield strength between 200 MPa and 500 MPa [70]. Results of compression testing of Ti–42Nb alloy manufactured by E-PBF showed at least 379 MPa yield strength, which is quite good and meets the requirements.

#### 3.2.3. Tests with the Ultrasonic Pulse-Echo Technique 

The non-destructive UPT was utilized to assess the properties of the alloy. This technique is widely acknowledged as one of the high precision methods for determining material properties, and it provides both longitudinal and shear elastic constants, while nanoindentation only provides longitudinal data [71,72]. The velocity of ultrasonic waves in the material is related to its elastic properties, and these can be used to estimate the parameters such as *E_u_*, *B*, ν, and Debye temperature (θ*_D_*). Corresponding properties obtained from the pulse-echo experiments with the tested specimens are listed in Table 5. It should be noted that utilized Ti–42Nb alloy is polycrystalline and corresponding calculations disregarded the presence of α″-martensite phase inclusions.

Obtained elastic constants *C*_11_, *C*_12_, and *C*_44_ satisfy the Born elastic stability conditions for cubic isotropy, indicating that the structure is stable [73]. These results are consistent with the values previously reported in the literature, as presented in Table 6. 

The elastic constants provide significant information about the physical properties of a material. For example, *C*_11_ represents the material’s resistance to linear compression under uniaxial stress in the *a*-direction, while *C*_44_ characterizes the resistance to shearing stress on the (100) plane along the [001] direction. Experimentally derived bulk modulus of Ti–42Nb specimens, which quantifies the resistance to volume change under hydrostatic pressure, is close to the values reported for similar composition alloys and exceeds the value obtained for pure titanium by other ultrasonic techniques. The obtained shear modulus, which characterizes the resistance to reversible and shear stress, is higher than the value obtained for pure titanium and agrees with the values reported for other β-alloys. The Young’s modulus of the Ti–42Nb wt.%, alloy (equivalent to Ti–27Nb at.%), is comparable to that reported in the literature for SLM-manufactured Ti–26 at.% Nb [50]. The Poisson’s ratio of 0.38 falls within the range of 1.0 to 0.5, indicating phase stability [74]. According to the literature, a Poisson’s ratio above 0.26 indicates ductile behavior of the material [75,76], which is supported by the low measured hardness value (2.28 GPa) compared to values obtained for similar materials using different non-acoustic techniques [77,78]. This conclusion is further supported by the relatively low Debye temperature, which is dependent on the bonding strength and is a certain measure of material hardness [74]. In addition, minimum thermal conductivity (*k_min_*), the fundamental characteristic for the new material was calculated.

Basing on the obtained results, Ti–42Nb alloy manufactured by E-PBF with chosen process parameters is ductile with reduced stiffness and hardness. It is worth noting that the value of *E_u_* obtained using UPT is 11% higher, while *H_u_* is 29% lower than those obtained using the nanoindentation method. These differences are likely due to the material’s anisotropy and the characteristics of the techniques [72]. The ultrasonic pulse-echo method enables the measurement of material properties throughout the entire volume averaging the elastic properties over a large number of grains along all crystal orientations, whereas nanoindentation produces spatially-localized measurements. Furthermore, the presence of the martensitic phase may also have an impact as the system is not entirely homogeneous.

**Table 6:** Elastic constants for different titanium alloys from literature, all in GPa unless shown otherwise.

Material	*C*_11_	*C*_12_	*C*_44_	ν	*B*	*E*	*G*	θ*_D_*	Method	Ref.
Ti–42 wt.% Nb	147.57	90.55	28.51	0.380	109.56	78.69	28.51	209.31	The ultrasonic pulse-echo method	This study
Ti–40 wt.% Nb	144	119	33	–	–		–	–	The resonant ultrasound spectroscopy	[79]
β-Ti_70_Nb_30_	137	110	33	0.41	–	66	–	–	The ultrasonic pulse-echo method	[52]
Ti–40 at.% Nb	157	112	40	–	127	–	26	–	The ultrasonic pulse-echo method	[80]
Ti–25 at.% Nb	117	106	20	–	109	35	12	–	The method of the ultrasoft pseudopotential method	[48]
Ti–26 at.% Nb (ingot)	–	–	–	0.3–7	–	70	25	–	The ultrasonic pulse-echo method	[50]
Ti–26 at.% Nb (SLM in situ)	–	–	–	0.35	–	78	29	–	The ultrasonic pulse-echo method	[50]
cp-Ti	98	83	38	–	88	–	–	264	Electromagnetic acoustic resonance for noncontracting excitation and detection.	[81]
cp-Ti (bar)	–	–	–	0.27	–	116	46	–	The ultrasonic pulse-echo method	[50]
β-Ti–45Nb	–	–	–	–	–	64	–	–	The ultrasonic pulse-echo method	[71]
β-Ti–7Mo–3Cr	–	–	–	–	–	–	36	–	The ultrasonic pulse-echo method	[70]
β-Ti–35Nb–5.7Ta–7.2Zr	–	–	–	–	–	5760	–	–	Nano-indentation and the ultrasonic pulse-echo method	[49]

### 3.3. Corrosion Resistance

**Figure 14:**
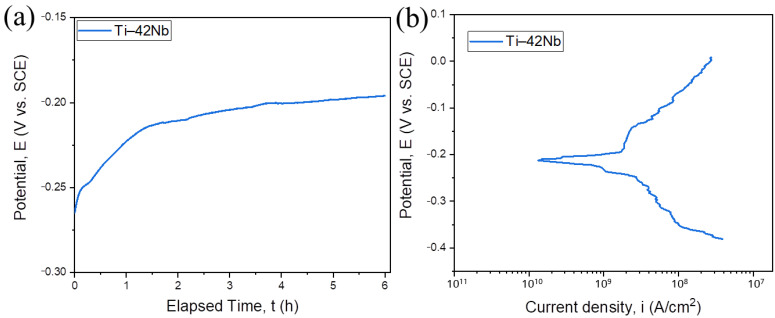
Evolution of OCP of Ti–42Nb alloy in SPB (**a**), Tafel plots of Ti–42Nb alloy in SPB (**b**).

The typical evolution of OCP of studied Ti–42Nb specimens is shown in Figure 14a, and the Tafel curves are depicted in Figure 14b.

Open circuit potential was monitored during the 6 h of immersion in SBF. Right from early stage of immersion, the OCP potential shifts toward more electropositive values (Figure 14a). This fact indicates that naturally air-passivated surfaces of the material were continuously further passivated in the SBF and the thickness of oxide film was growing [14]. After 3 h the OCP potential has a tendency to stabilize, although still slightly increasing up to the end of the test at 6 h. The main corrosion parameters determined through Tafel measurements are summarized in Table 7. Based on the Stern-Geary relationship (Equation (2)) the polarization resistance *R_p_* was calculated:

(2) $$R_{p}=\frac{1}{2{.3i}_{corr}}\frac{\beta_{a\;}\left|\beta_{c\;}\right|}{\beta_{a\;}+\left|\beta_{c\;}\right|},$$

where $i_{corr}$ is corrosion current density, $\beta_{a\;}$ is anodic Tafel slope, and $\beta_{c\;}$ is cathodic Tafel slope.

The measured corrosion resistance of a material is defined by such parameters as temperature, chemical composition and pH of electrolyte and technique used. Moreover, some important surface properties like composition, microstructure and roughness influence the anticorrosion properties of the samples. Although available literature data do not allow full comparison with the measured ones, one can still make certain conclusions. It can be observed that reported corrosion potential values fall within the range between −614 and −151 mV. The value of the *E_corr_* obtained from the electrochemical tests on Ti–42Nb is −252 mV which is within reported range but closer to more electropositive values reported in the literature, thus demonstrating the ability of the alloy to adequately resist corrosion in SBF. The value of *i_corr_* obtained for the Ti–42Nb alloy in present study (3.84 nA/cm^2^) is near to the lowest value reported in the literature, thus demonstrating a better corrosion resistance compared to the Ti–Nb alloys investigated previously. From the polarization point of view, the Ti–42Nb registered quite high *R_p_* value (20.91 MΩ·cm^2^) suggesting that the oxide formed on the surface plays the role of a very good kinetic barrier. The composition of alloy affects the structure and stability of the oxide on the surface, which is important for metallic implants. Wang et al. [83] studied the corrosion resistance of Ti alloys with different Nb content (15, 32 and 38 wt.%) suggesting that the high corrosion current density may be related to the presence of α″-phase. Taking into account all abovementioned aspects it can be concluded that Ti–42Nb alloy has an excellent corrosion resistance in the SBF.

A more electropositive value of corrosion potential (*E_corr_*), a small value of corrosion current density and a high polarization resistance point out to an overall good corrosion resistance of a material. The Ti alloys are well known for their very good anticorrosive properties. Currently, new alloys are being developed which, in addition to other designed properties, have also an enhanced corrosion resistance [14]. In the literature various alloys of Ti with Nb are investigated for corrosion resistance and the main corrosion parameters determined in such experiments reported in the literature are summarized in Table 8.

## 4. Conclusions

To ensure the success of medical implants, it is vital to carefully consider the mechanical, physical, chemical, and biological characteristics of the biomaterials being used. In our previous work process parameters for E-PBF manufacturing of Ti–42Nb alloy have been selected. In this study the Ti–42Nb alloy produced using the regime providing dense samples, underwent a thorough evaluation of its mechanical and electrochemical properties. Based on these following key findings can be formulated:

1. Microstructure investigations by XRD and TEM of the samples cut both along the BD and in layer plane confirm the domination of β-phase. However, the presence of α″-phase was also demonstrated, although the impact of this phase on mechanical properties and corrosion resistance of β alloys is quite challenging to evaluate. SEM and EBSD investigations revealed columnar grains typical for AM alloys. However, expected anisotropy of mechanical properties was not found. It can be attributed to the domination of relatively symmetric equiaxed β-grains with a weak texture. This leads to a reduced anisotropy of mechanical properties.
2. According to the results of nanoindentation testing, the structure the alloy has slightly varying properties that are influenced by the E-PBF method features. The investigation of hardness and elastic modulus yielded average values of 3.2 ± 0.5 GPa and 89.9 ± 8.3 GPa, respectively. Compression mechanical testing showed that the chosen alloy meets the requirements for supporting human bones.
3. The UPT was used to calculate such values as the elastic constants and moduli (*E* = 78.69 GPa), as well as the Debye temperature and minimum thermal conductivity. Such values for Ti–Nb binary alloys were not published before. The UPT results, as well as other methods, confirmed the ductile nature of the alloy.
4. The values of the modulus of elasticity and hardness obtained by different methods correlate with the literature ones. Observed differences can be attributed to the material’s anisotropy and the characteristics of the specific techniques used in different studies.
5. XPS results confirmed the dominant presence of TiO_2_ and Nb_2_O_5_ on the alloy’s surface. Moreover, the small amount of NbC, associated with the initial powder composition, was also found. Further development of an oxide layer on the surface of the Ti–42Nb alloy in SBF resulted in excellent corrosion resistance, as demonstrated by the changes in the OCP and Tafel plot.

In conclusion, it is worth noting, that nanoindentation is an area-dependent method and more dense indentations should be carried out for more accurate results. Additional methods, such as used ultrasonic pulse-echo one, allow for higher fidelity of acquired results by comparing values from the methods with high spatial resolutions (like nanoindentation) and the ones producing the values averaged through the bulk (like UPT). 

Results from employed techniques allows to conclude that Ti–42 wt.% Nb alloy obtained by the E-PBF method has low modulus of elasticity, acceptable yield strength, and high corrosion properties and is a promising biomaterial for implant manufacturing. 

This study expands the possibilities of using the E-PBF method and suggests the use of the resulting parameter window to produce Ti–Nb alloys with different niobium content, as well as porous scaffolds with different topologies. 

## Figures and Tables

**Table 1:** The set of parameters for fabrication.

*d* (µm)	*I* (mA)	*v* (mm/s)	*h* (µm)	Line Energy (J/mm)	Area Energy (J/mm^2^)
100	4.00	800	100	0.3	3

**Table 2:** Chemical composition of SBF.

No.	Composition	Concentration (g/L)
1	NaCl	8.035
2	NaHCO_3_	0.355
3	KCl	0.225
4	K_2_HPO_4_ × 3H_2_O	0.231
5	MgCl_2_ × 6H_2_O	0.311
6	1 M HCl	3.2 mL
7	CaCl_2_	0.292
8	Na_2_SO_4_	0.072
9	(HOCH_2_)_3_CNH_2_ (Tris)	6.118

**Table 3:** Binding energies of elements and their relative atom concentration from XPS survey spectra.

Element	Position, eV	FWHM	Concentration, at.%
O 1s	530	3.22	40.5
C 1s	285	2.84	46.9
Ti 2p	458	2.23	7.4
Nb 3d	207	4.67	4.7
N 1s	400	2.01	0.6

**Table 4:** Calculated parameters obtained from X-ray diffraction patterns.

Material	*a* (Å)	*D* (nm)	δ·10^−3^ (nm^−2^)	ε·10^−3^
Ti–42Nb	3.290	22.71	2.401	2.301

**Table 5:** Ultrasonically-measured mechanical properties of E-PBF manufactured Ti–42Nb samples. All values are in GPa unless shown otherwise.

Material	*v_L_* (m·s^−1^)	*v_T_* (m·s^−1^)	*v_m_* (m·s^−1^)	*M* (g·mol^−1^)	ν	*G*	*B*
Ti–42Nb	5315.44	2348.85	2651.76	60.10	0.380	28.51	109.56
	***H_u_***	***E_u_***	***C*_11_**	***C*_12_**	***C*_44_**	**θ*_D_* (K)**	***k*_min_ (W·(m·K)^−1^)**
	2.28	78.69	147.57	90.55	28.51	209.31	4.98

**Table 7:** Main corrosion parameters of Ti–42Nb alloy in SBF.

Material	*E_corr_* (mV)	*i_corr_* (nA/cm^2^)	β*_a_* (mV)	β*_c_* (mV)	*R_p_* (MΩ·cm^2^)
Ti–42Nb	–225 ± 2	3.84 ± 0.02	279.19 ± 4.21	524.01 ± 3.33	20.91 ± 0.94

**Table 8:** Corrosion parameters for different Ti–Nb alloys from literature.

Alloy	Electrolyte	*E_corr_* (mV)	*i_corr_* (nA/cm^2^)	*R_p_* (kΩ·cm^2^)	Reference
Ti–42 wt.% Nb	SBF	–225	3.84	20.91·10^3^	this study
Ti–20 wt.% Nb	0.9% NaCl	–520	470	–	[23]
Ti–16 at.% Nb	Hank’s solution	–403	3.557	–	[39]
Ti–40 wt.% Nb	0.9% NaCl	–285	–	–	[21]
Ti–40 wt.% Nb	0.9% NaCl	–360	–	–	[19]
Ti–45 wt.% Nb	acidified saliva	–614	85	336	[20]
	fluoridated saliva	–560	181	323	
Ti–30 at.% Nb	SBF	–151	91.9	1.844	[82]
Ti–38 wt.% Nb	Ringer’s solution	–365	320	–	[83]
Ti–16 wt.% Nb	SBF	–424	275	–	[84]
Ti–40 wt.% Nb	SBF	–389	296	–	[85]

## Data Availability

The data presented in this study are available on request from the corresponding author.
